# Our Food: Packaging & Public Health

**DOI:** 10.1289/ehp.120-a232

**Published:** 2012-06-01

**Authors:** Luz Claudio

**Affiliations:** Luz Claudio, PhD, is a tenured associate professor of preventive medicine at Mount Sinai School of Medicine, where she is director of International Health.


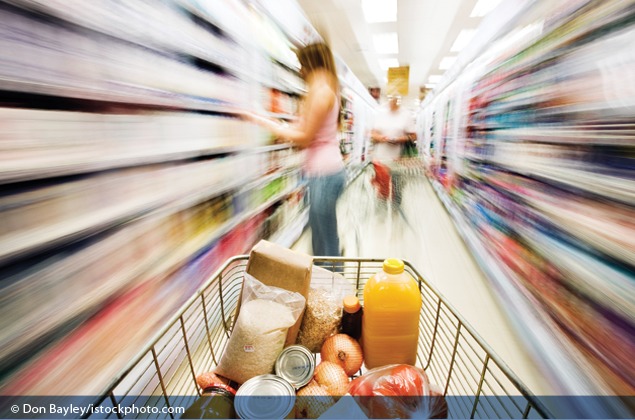
Your daily routine has many close encounters with food packaging: For breakfast, cereal from a paperboard box and a can of energy drink. For lunch, canned tuna and a plastic bottle of water. Afternoon snack, a foil-lined plastic bag of potato chips and a shrink-wrapped tray of fruit. By the time you dish up your supper of baked chicken and frozen broccoli, you’ve reaped the benefits of—and discarded—numerous different food-packaging materials. “Packaged food is very convenient. It is nice to have good food that you can grab and go,” says Claudia DeMegret, director of education at the City Parks Foundation in New York. “You try to be conscientious—buy fresh food and recycle. But you also have to wonder about how all this packaging affects the food we feed our kids and . . . how much of it ends up in landfills.”

Food packaging does much more than simply hold a product. It keeps food safe and fresh, tells us how to safely store and prepare it, displays barcodes that facilitate purchasing, provides nutritional information, and protects products during transport, delivery, and storage. On the other hand, packaging also fills trash containers and landfills, lasting far longer than the products it was made to contain. It consumes natural resources. And it can also transfer chemicals into our food, with unknown health effects. Our relationship with packaging—you could say it’s complicated.

## A History of Benefits

For millennia, humans stored their food in containers they found in nature—dried gourds, shells, hollow logs, leaves—as well as baskets and pottery. By the first century BC, the Chinese were wrapping foods with treated tree bark and other forerunners of paper. Centuries later, Napoleon Bonaparte used some of the first mass-produced canned food to feed his troops in the Franco-Austrian War of 1809. Plastics were discovered in the decades following that innovation but were not used beyond military purposes until well into the twentieth century.^^1^^^^,^^^^2^^

The art and science of food packaging have evolved a long way from those origins. Today, products often are wrapped in multiple layers of packaging to get them safely from the point of manufacture to consumers’ cupboards and refrigerators. Food packaging can improve food safety by alleviating bacterial contamination. It has been proposed that increased use of packaging for fresh produce could prevent contamination with *Salmonella* spp., a leading cause of foodborne diseases.^^3^^

“We appreciate foodservice packaging because of the convenience it affords for our busy lifestyles, but often we forget about the main benefit: sanitation,” says Lynn Dyer, president of the Foodservice Packaging Institute, an industry association. “That’s why single-use products were invented over 100 years ago—to help stop the spread of contagious diseases.”

In addition to preventing bacterial contamination, food packaging also extends the shelf life of products, which allows for broader distribution and reduced food waste. Food waste is a significant problem in the United States. The Environ-mental Protection Agency (EPA) estimates that 34 million tons of food was thrown away in 2010, representing close to 14% of the municipal solid waste generated in the United States.^^4^^ (Ironically, using more packaging to reduce food waste creates another waste problem: In 2010 household packaging constituted almost one-third of the municipal solid waste generated.^^5^^)

In the United States, all food-contact substances (FCSs)—defined as substances “intended for use as a component of materi-als used in manufacturing, packing, packaging, transporting, or holding food if such use is not intended to have any technical effect in such food”^^6^^—are regulated by the Food and Drug Administration (FDA). Different packaging materials offer different advantages. Glass preserves taste well and is chemically inert. Paper and paperboard are economic to produce and easy to print on. They are also lightweight, which reduces the fuel used for the transport of goods.^^7^^ Steel and aluminum offer the advantages of malleability, impermeability, and ease of recycling. Aluminum can also be bound to paper or plastic films for more versatility in the types of packaging that can be produced. And plastics have revolutionized the packaging industry because they are highly moldable into infinite shapes, lightweight, inexpensive, easy to seal, and durable.

## Potential Chemical Exposures from Packaging

It is well known that chemical components from packaging can migrate into foods, but questions of how much migration occurs and what the potential health effects may be are gaining more attention from researchers and regulators.^^8^^ However, few studies to date have looked at adverse human health effects of these exposures.

Different types of packaging materials pose different potential chemical exposures. Glass, for instance, is generally recognized as safe by the FDA when used as a container for holding food. But some glass bottles and jars may contain lead. Researchers at the Institute of Environmental Geochemistry of the University of Heidelberg in Germany assessed 125 brands of drinking water from 28 countries and showed that waters packaged in glass bottles contained 26–57 times more lead than comparable waters bottled in polyethylene terephthalate (PET) plastic. The increased lead content appeared to be a result of leaching from glass containers, although at < 1–761 ng/L, even the highest lead levels detected were well below maximum allowances for drinking water (10 µg/L in the European Union and Can-ada, and 15 µg/L in the United States).^^9^^

Other studies have found chemical contamination of food coming not from glass itself but from materials used to seal the metal lids on glass jars. In work by a Danish group, some foods in glass jars sealed with polyvinyl chloride (PVC) gaskets were found to contain di(2-ethylhexyl)phthalate (DEHP) and other phthalates at levels deemed unacceptable by the European Food Safety Authority.^^10^^^^,^^^^11^^ These studies did not assess potential health effects from this exposure, but in other studies phthalates have been associated with endocrine disruption in humans.^^12^^^^,^^^^13^^^^,^^^^14^^

Environmental health concerns associ-ated with the use of paper food packaging have focused on the use of recycled paper products. Printing inks from earlier incarnations of the paper can be trapped in this material, potentially exposing consumers to phthalates as well as to other suspected endocrine disruptors, including benzophenones and mineral oils.^^8^^ A study conducted by a German group showed that infant foods packed in recycled paperboard boxes with coated paper liners were contaminated with diisobutyl phthalate and di-*n*-butyl phthalate, with a few samples containing the former at levels exceeding European Commission limits for food contaminants.^^15^^ The authors noted that inner liners made of aluminum-coated foil were much more effective than coated paper at blocking the migration of phthalates from recycled paperboard packaging.

There also have been problems with the liners themselves in some paper boxes. In 2010 Kellogg Company recalled 28 million boxes of cereal because of elevated levels of methylnaphthalene^^16^^ that leached from the coated paper lining the boxes.^^17^^ Although the potential consequences of ingestion of this compound are not well understood, at least five consumers reportedly became ill after eating the contaminated cereal.^^18^^

Perhaps the hottest current debate regarding food packaging is the use of epoxy-based resins containing bisphenol A (BPA) in metal can liners (BPA is also used in hard, clear polycarbonate plastic).^^19^^ In 2008 the Nat-ional Toxicology Program released a review of the evidence on the toxicity of BPA expressing “some concern” that the compound may adversely affect the brain and prostate gland in fetuses, infants, and children at exposure levels documented in the general U.S. population.^^20^^ The Natural Resources Defense Council has petitioned the FDA to ban the use of BPA in food packaging, but on 30 March 2012 the FDA issued an interim ruling denying that request, pending further research.^^21^^ Currently the FDA allows the use of BPA in food-contact applications.

In one Texas-based study of BPA in packaged foods, researchers assessed 105 samples of fresh, plastic-wrapped, and canned foods, and found detectable levels of the chemical in 60% of them (including some of the fresh foods).^^22^^ The researchers calculated BPA intake for adults and children eating regular servings of some of the foods sampled. Their estimates fell between the reference doses established by the European Commission Scientific Committee on Food Safety (10 µg/kg/day) and the U.S. EPA (50 µg/kg/day). Despite the relatively low estimated doses from eating any one food, these authors and others^^8^^^^,^^^^23^^ point out there are multiple sources of intake of BPA, and evidence increasingly suggests that BPA and other endocrine disruptors—like the hormones they mimic—may cause unexpected effects even at tiny doses, although the extent to which these effects may occur in humans is still under investigation.^^24^^

**Figure:**
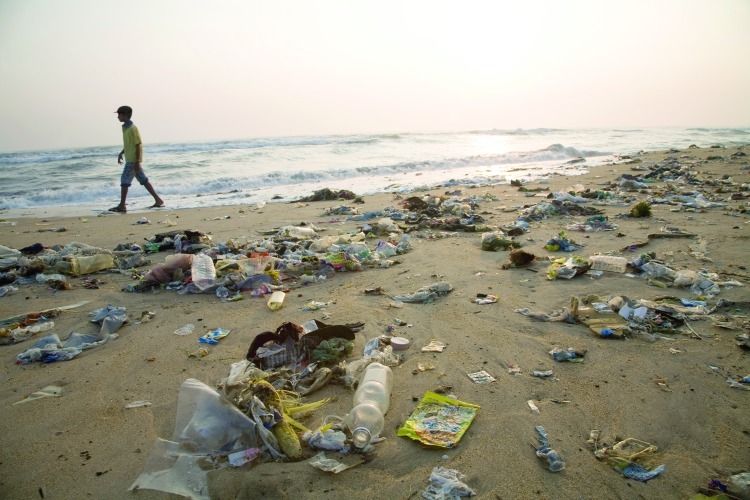
**Packaging in the Sea** Any food packaging that is not recycled or properly disposed of is likely to end up as litter. Since 1986 volunteers with the Ocean Conservancy’s annual one-day International Coastal Cleanups have picked up tens of millions of food-packaging items from beaches around the world.^29^ Other debris makes its way into oceans, perhaps most notoriously ending up as part of the Great Pacific Garbage Patch and other accumulations of trash formed by converging ocean currents. A study released 9 May 2012 estimates plastic contamination in the Great Pacific Garbage Patch has increased by two orders of magnitude since 1972.^30^ Although these patches can contain large chunks of debris, they consist primarily of microscopic weathered particles of plastic and other materials, forming a sort of “trash soup” that is difficult to quantify and clean up.^29^ Charles J. Moore, founder of the Algalita Marine Research Foundation and one of the first people to document waste contamination of the North Pacific Central Gyre,^31^ says this soup likely finds its way up the food chain as it mixes with the plankton consumed by fish. It is unclear whether ingesting microplastic particles causes any adverse health effects anywhere in the food chain, although there is evidence these particles may bind relatively large amounts of persistent organic pollutants found in seawater, then release them into marine organisms, with unknown effects.^32^ © John Lund/Getty Images

Some chemicals of concern, such as phthalates, have been phased out of use in food packaging. For instance, the American Plastics Council has stated that “phthalates are not used in plastic beverage bottles, nor are they used in plastic food wrap, food containers, or any other type of plastic food packaging sold in the United States.”^^25^^ Steve Russell, vice president of the Plastics Division of the American Chemistry Council, says that in the United States very little PVC is used in food contact except for meat and cling wrap, and in that application, phthalates have been replaced with alternative plasticizers such as di-(2-ethylhexyl) adipate. Adipates have been shown to potentially leach into foods, and their effects are being studied in laboratory animals, but effects on humans—if any—are not known.^^26^^^^,^^^^27^^

## Room for Improvement

Although food packaging is important for sanitation and convenience, studies such as these point to the need for a better understanding of the scope and impact of chemical contamination of food via packaging. In a 2007 review of packaging contaminants in European food, Koni Grob and colleagues of the Official Food Control Authority of Canton of Zürich, Switzerland, estimated that migration of contaminants from food packaging may greatly exceed that of other contaminants, such as pesticides and environmental pollutants. “In terms of amounts,” the authors wrote, “migration from packaging material is the most important source: it exceeds most others by a factor of 100–1000.”^^28^^

Although the authors noted these amounts “measure the degree of contamination and are not indicative of risks,” they further point out, “Legal limits for migration from packaging materials are high: the global migration limit sanctions a contamination which is unparalleled, and restrictions for specific components . . . probably [do] not correspond to the expectations of the consumers.”^^28^^

It is difficult to estimate the risk of chronic ingestion of contaminants from food packaging, as so little is known. It is even more difficult, at this point, to estimate any public-health impact that might result from that ingestion or to weigh the potential negative impacts against the known benefits related to reduced spoilage and microbial contamination.

**Figure:**
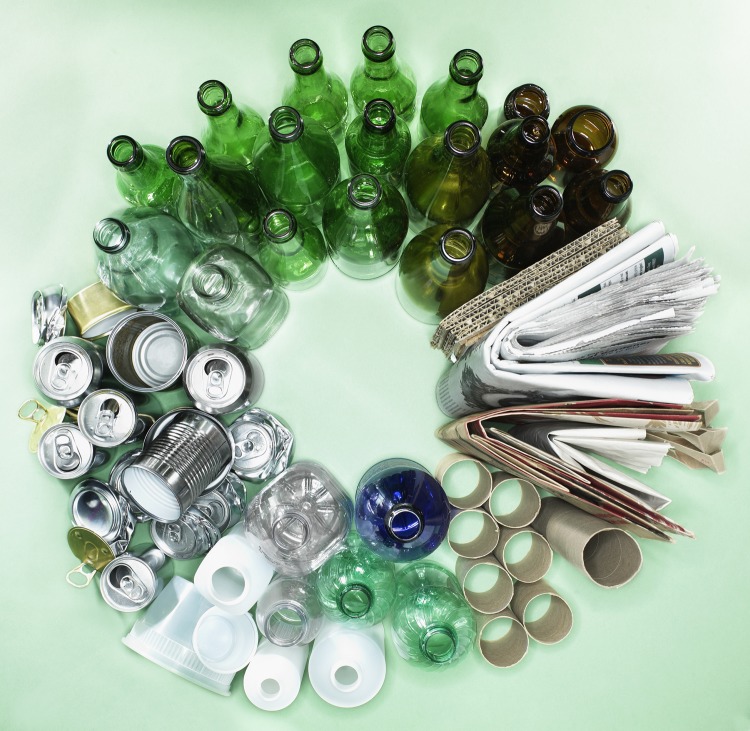
**What Is Being Done to Address Environmental Impacts?** Efforts to address the environmental impacts of packaging include those that aim for source reduction, reuse, and recycling. **Reduce:** Source reduction can be achieved by “lightweighting,” or using less material to make the same packaging. Glass containers have decreased in weight by nearly 50% in 10 years, and between the 1970s and 2000s, two-liter PET soft-drink bottles got 25% lighter, aluminum cans got 26% lighter, and steel cans and plastic grocery sacks each lightened up by 40%.^33^^,^^34^ Another form of lightweighting is the use of pouches made of a thin film of plastic combined with other materials. The Swedish packaging developer Ecolean produces a one-liter pouch that weighs only 16 grams, nearly half as much as a one-liter polyethylene terephthalate (PET) bottle.^35^ **Reuse:** Reusable and refillable containers are another way in which companies can implement source reduction. Although refillable milk bottles are no longer common in the United States, they are still used in some areas of Britain where milk production is local. In Germany, about half the soft drinks and mineral water and most of the beer is sold in refillable bottles.^36^ **Recycle:** Recovery for recycling is encouraged by beverage container laws, also known as “bottle bills,” in which a cash deposit of 5–10¢ is added to the product and reimbursed when the empty container is redeemed. Currently only 10 U.S. states have such laws in place—California, Connecticut, Hawaii, Iowa, Maine, Massachusetts, Michigan, New York, Oregon, and Vermont (unclaimed deposits, which can amount to millions of dollars per year, revert to the state and/or bottlers and distributors).^37^ According to the nonprofit Container Recycling Institute, states that do not have bottle bills have a beverage-container recycling rate of about 24%, whereas states with bottle bills recycle about 60% of their containers.^38^ Glass can be recycled endlessly with little loss of quality or purity of the material. The demand for glass for recycling exceeds supply, with only 33% of discarded glass bottles and jars actually recovered for recycling in 2010. Paper food packaging is one of the least recycled materials, with 25% of discarded cartons, boxes, and bags recovered for recycling the same year. Steel cans were the most highly recycled metal food packaging material at 67% recovery, followed by aluminum cans at 50% recovery. Just under 30% of PET and high-density polyethylene (HDPE) containers were recovered.^5^ Although most food-packaging plastics can, in theory, be melted to make new products, some are easier and cheaper to collect and process than others, and the demand for recycled plastics differs by material, according to Steve Russell, vice president of the Plastics Division of the American Chemistry Council. Metallized plastics and laminates such as those used in juice pouches are difficult to recycle because of the mixtures of materials used. However, TerraCycle, a Trenton, New Jersey–based recycling company, collects these and other types of hard-to-recycle waste and “upcycles” them—that is, uses them to create new and innovative household and personal items.^39^ Recycled material may not be of the same quality or purity as the original raw material, or additional steps may be necessary to achieve the quality or purity needed for the next use of the material. For instance, plastics containing additives to help them degrade may be unsuitable as food-contact substances in their next life if any of the degradable additives remain after recycling, says Russell. The FDA therefore must preapprove any recycled materials intended to be used in contact with food.^40^ There’s much more to recycling than reducing the waste stream, however. “The main concern with large volumes of packaging waste is not that we are filling up landfills, it is that we are squandering materials,” says Mathy Stanislaus, assistant administrator for the EPA Office of Solid Waste and Emergency Response. For instance, in 2006 about 331 million barrels of petroleum and natural gas were used to make plastic materials in the United States, representing 4.6% of total U.S. petroleum consumption that year.^41^ “When we fail to find better ways to reduce, reuse, or recycle [packaging] materials, then we must use new materials,” Stanislaus says, “and that has significant negative impacts on human health and the environment.” © Ryan McVay/Getty Images

But the need for more research is clear. “While pesticides are thoroughly evaluated and well controlled in their use, only a small fraction of the substances migrating from food packaging have been evaluated—less than fifteen hundred—and the majority have not even been identified,” Grob says. “If fifty to a hundred thousand sub-stances migrate [from packaging into foods] at levels sometimes exceeding the threshold of toxicological concern, and if one out of a hundred substances harms our health, this is likely to cause serious damage.”

*Editor’s note: Innovations in packaging materials and processes are being developed that use alternative materials to address the migration of potentially toxic chemicals into foods. Others address the volume of food-packaging trash by incorporating biodegradable components.* EHP *will explore food-packaging innovations in an upcoming issue.*

## References

[1] Berger KR (2005). A Brief History of Packaging.. Gainesville, FL:Agricultural and Biological Engineering Department, Florida Cooperative Extension Service, Institute of Food and Agricultural Sciences, University of Florida.

[2] Risch SJ (2009). Food packaging history and innovations.. J Agric Food Chem.

[3] Hanning IB (2009). Salmonellosis outbreaks in the United States due to fresh produce: sources and potential intervention measures.. Foodborne Path Dis.

[4] EPA. Basic Information about Food Waste [website]. Washington, DC:U.S. Environmental Protection Agency (updated 9 Apr 2012). Available: http://www.epa.gov/osw/conserve/materials/organics/food/fd-basic.htm [accessed 7 May 2012].

[5] EPA. Municipal Solid Waste Generation, Recycling and Disposal in the United States: Tables and Figures for 2010. Washington, DC:U.S. Environmental Protection Agency (Dec 2011). Available: http://www.epa.gov/wastes/nonhaz/municipal/pubs/2010_MSW_Tables_and_Figures_508.pdf [accessed 7 May 2012].

[6] GPO. U.S. Code, Title 21, Food and Drugs. Chapter 9, Subchapter IV, §348 (h) 6. Washington, DC:U.S. Government Printing Office. Available: http://www.gpo.gov/fdsys/pkg/USCODE-2010-title21/html/USCODE-2010-title21-chap9-subchapIV-sec348.htm [accessed 7 May 2012].

[7] Robertson GL (2005). Food Packaging: Principles and Practice, 2nd ed.. Boca Raton, FL:CRC Press.

[8] Muncke J. (2011). Endocrine disrupting chemicals and other substances of concern in food contact materials: an updated review of exposure, effect and risk assessment.. J Steroid Biochem Molec Biol.

[9] Shotyk W, Krachler M. (2007). Lead in bottled waters: contamination from glass and comparison with pristine groundwater.. Environ Sci Technol.

[10] Petersen JH, Jensen LK (2010). Phthalates and food-contact materials: enforcing the 2008 European Union plastics legislation.. Food Addit Contam: Part A: Chem Anal Control Expo Risk Assess.

[11] Pederson GA (2008). Migration of epoxidized soybean oil (ESBO) and phthalates from twist closures into food and enforcement of the overall migration limit.. Food Addit Contam Part A Chem Anal Control Expo Risk Assess.

[12] Duty SM (2003). The relationship between environmental exposures to phthalates and DNA damage in human sperm using the neutral comet assay.. Environ Health Perspect.

[13] Swan SH (2005). Decrease in anogenital distance among male infants with prenatal phthalate exposure.. Environ Health Perspect.

[14] Latini G (2004). Di-2-ethylhexyl phthalate and endocrine disruption: a review.. Curr Drug Targets: Immune Endocr Metabol Disord.

[15] Gärtner S (2009). Analysis and migration of phthalates in infant food packed in recycled paperboard.. J Agric Food Chem.

[16] Lunder S. Kellogg’s Cereal Recall: Health Risks from Packaging? Washington, DC:Environmental Working Group (12 Jul 2010). Available: http://www.ewg.org/health-risks-from-packaging [accessed 7 May 2012].

[17] Harrington R. Kellogg Issues Massive Recall as Tainted Packaging Sparks Health Fears. FoodProductionDaily.com (28 Jun 2010). Available: http://www.foodproductiondaily.com/Quality-Safety/Kellogg-issues-massive-recall-as-tainted-packaging-sparks-health-fears [accessed 7 May 2012].

[18] Brat I, Becker N. Kellogg Recalls Cereal. The Wall Street Journal, Business section, online edition (26 Jun 2010). Available: http://online.wsj.com/article/SB10001424052748703615104575328883385848118.html [accessed 7 May 2012].

[19] Vandenberg LN (2009). Bisphenol-A and the great divide: a review of controversies in the field of endocrine disruption.. Endocrinol Rev.

[20] NTP. NTP-CERHR Monograph on the Potential Human Reproductive and Developmental Effects of Bisphenol-A. Research Triangle Park, NC:Center for the Evaluation of Risks to Human Reproduction, National Toxicology Program, U.S. Department of Health and Human Services (Sep 2008). Available: http://ntp.niehs.nih.gov/ntp/ohat/bisphenol/bisphenol.pdf [accessed 7 May 2012].

[21] FDA. Bisphenol A (BPA): Use in Food Contact Application [website]. Silver Spring, MD:U.S. Food and Drug Administration (updated 30 Mar 2012). Available: http://www.fda.gov/NewsEvents/PublicHealthFocus/ucm064437.htm [accessed 7 May 2012].

[22] Schecter A (2010). Bisphenol A (BPA) in U.S. food.. Environ Sci Technol.

[23] Muncke J. (2009). Exposure to endocrine disrupting compounds via the food chain: is packaging a relevant source?. Sci Total Environ.

[24] Ahearn A. Low-dose exposure to endocrine disruptors, with Laura Vandenberg [podcast]. Environ Health Perspect; http://dx.doi.org/10.1289/ehp.trp060112 [online 1 Jun 2012]. 22833914

[25] Enneking PA Phthalates not in plastic food packaging.. Environ Health Perspect.

[26] ter Veld MGR (2009). Estrogenicity of food-associated estrogenic compounds in the fetuses of female transgenic mice upon oral and IP maternal exposure.. Reprod Toxicol.

[27] Jarfelt K Antiandrogenic effects in male rats perinatally exposed to a mixture of di(2-ethylhexyl) phthalate and di(2-ethylhexyl) adipate.. Reprod Toxicol.

[28] Grob K (2006). Food contamination with organic materials in perspective: packaging materials as the largest and least controlled source? A view focusing on the European situation.. Crit Rev Food Sci Nutr.

[29] Ocean Conservancy. Tracking Trash: 25 Years of Action for the Ocean. Washington, DC:Ocean Conservancy (2011). Available: http://act.oceanconservancy.org/pdf/Marine_Debris_2011_Report_OC.pdf [accessed 7 May 2012].

[30] Goldstein MC, et al. Increased oceanic microplastic debris enhances oviposition in an endemic pelagic insect. Biol Lett; http://dx.doi.org/10.1098/rsbl.2012.0298 [online 7 May 2012]. 10.1098/rsbl.2012.0298 PMC3440973 22573831

[31] Moore CJ (2001). A comparison of plastic and plankton in the North Pacific Central Gyre.. Mar Pollut Bull.

[32] Gorycka M. Environmental Risks of Microplastics. Amsterdam, the Netherlands:Vrije Universiteit (6 Jul 2009). Available: http://www.cleanup-sa.co.za/Images/Environmental_Risks_Microplastics.pdf [accessed 7 May 2012].

[33] Marsh K, Bugusu B. (2007). Food packaging—roles, materials, and environmental issues.. J Food Sci.

[34] EPIC. The Invisible “R” Reduction. Mississauga, Canada:Environment and Plastics Industry Council. Available: http://www.plastics.ca/_files/file.php?fileid=itemThReciXyTj&filename=file_files_Invisiblr_R.pdf [accessed 7 May 2012].

[35] Ecolean. Environmental Comparison, Ecolean Air [website]. Helsingborg, Sweden:Ecolean AB (2012). Available: http://www.ecolean.com/en/environment/environmental-comparison-ecolean-air/ [accessed 7 May 2012].

[36] INCPEN. Reuseable Packaging [fact sheet]. Reading, UK:Industry Council for Packaging and the Environment (2010). Available: http://www.incpen.org/displayarticle.asp?a=5&c=2 [accessed 7 May 2012].

[37] Bottle Bill Resource Guide [website]. Culver City, CA:Container Recycling Institute (2011). Available: http://www.bottlebill.org/ [accessed 7 May 2012].

[38] Bottle Bills [website]. Culver City, CA:Container Recycling Institute (2003–2013). Available: http://www.container-recycling.org/issues/bottlebills.htm [accessed 7 May 2012].

[39] Products Index [website]. Trenton, NJ:TerraCycle, Inc. (2012). Available: http://www.terracycle.net/en-US/products/page/1.html [accessed 7 May 2012].

[40] FDA. Submissions on Post-Consumer Recycled (PCR) Plastics for Food-Contact Articles [website]. Silver Spring, MD:U.S. Food and Drug Administration (updated 2 Mar 2012). Available: http://www.accessdata.fda.gov/scripts/fcn/fcnNavigation.cfm?rpt=recyListing&displayAll=true [accessed 7 May 2012].

[41] EIA. Frequently Asked Questions: How Much Oil Is Used to Make Plastic? [website]. Washington, DC:Energy Information Administration, U.S. Department of Energy (updated 27 May 2011). Available: http://205.254.135.7/tools/faqs/faq.cfm?id=34&t=6 [accessed 7 May 2012].

